# α-Synuclein deficiency promotes neuroinflammation by increasing Th1 cell-mediated immune responses

**DOI:** 10.1186/s12974-016-0694-4

**Published:** 2016-08-26

**Authors:** Benjamin Ettle, Kristina Kuhbandner, Stefanie Jörg, Alana Hoffmann, Jürgen Winkler, Ralf A. Linker

**Affiliations:** 1 Department of Molecular Neurology, University Hospital Erlangen, Friedrich-Alexander-University Erlangen-Nürnberg, Schwabachanlage 6, 91054 Erlangen, Germany; 2 Department of Neurology, University Hospital Erlangen, Friedrich-Alexander-University Erlangen-Nürnberg, Schwabachanlage 6, 91054 Erlangen, Germany

**Keywords:** α-Synuclein, Experimental autoimmune encephalomyelitis, Neuroinflammation, Multiple sclerosis

## Abstract

**Background:**

Increased α-synuclein immunoreactivity has been associated with inflammatory activity in multiple sclerosis (MS) lesions, but the function of α-synuclein in neuroinflammation remains unknown. The aim of this study was to examine the role of α-synuclein in immunological processes in murine experimental autoimmune encephalomyelitis (EAE) as a model of MS.

**Findings:**

We studied EAE in wildtype (aSyn^+/+^) and α-synuclein knockout (aSyn^−/−^) mice on a C57BL/6N background. In the spleen and spinal cord of aSyn^+/+^ mice, we observed a gradual reduction of α-synuclein expression during EAE, starting already in the pre-symptomatic disease phase. Compared to aSyn^+/+^ mice, aSyn^−/−^ mice showed an earlier onset of symptoms but no differences in symptom severity at the peak of disease. Earlier symptom onset was accompanied by increased spinal cord infiltration of CD4^+^ T cells, predominantly of interferon-γ-producing T helper 1 (Th1) cells, and reduced infiltration of regulatory T cells, whereas antigen-presenting cells were unaltered. Pre-symptomatically, aSyn^−/−^ mice exhibited hyperproliferative CD4^+^ splenocytes consistent with increased splenic interleukin-2 mRNA expression, resulting in increased numbers of Th1 cells in the spleen at the onset of symptoms.

**Conclusions:**

Our findings indicate a functional role of α-synuclein in early EAE by increasing Th1 cell-mediated immune response.

## Introduction

Multiple sclerosis (MS) and its murine model experimental autoimmune encephalomyelitis (EAE) are characterized by a primarily T cell-mediated autoimmune attack against oligodendrocytes and myelin of the central nervous system (CNS), subsequently leading to axonal degeneration and neuronal loss. However, factors that drive and modify the immune response in MS and EAE are still incompletely defined.

A candidate factor acting as modulator of inflammatory processes in MS and EAE is α-synuclein. Under physiological conditions, α-synuclein is predominantly expressed in neurons [1], but expression has also been observed in glia [1, 2] and hematopoietic cells such as T cells and monocytes [3, 4]. In human diseases, α-synuclein accumulates and forms insoluble fibrils as part of intraneuronal and intraoligodendroglial inclusions in chronic, age-related neurodegenerative diseases such as Parkinson’s disease (PD), dementia with Lewy bodies, and multiple system atrophy [5]. Importantly, peripheral and CNS inflammation aggravates degenerative features in these so-called synucleinopathies [6–8].

Given that increased α-synuclein immunoreactivity has been observed in microglia/macrophages within inflammatory-active MS lesions [9], α-synuclein may also be involved in the immune reaction that underlies degenerative changes in MS and EAE. To investigate this hypothesis, we employed EAE in wildtype (aSyn^+/+^) and α-synuclein knockout (aSyn^−/−^) mice, focusing on the pre-symptomatic and acute phase of EAE. Our data show an earlier onset of symptoms accompanied by increased T helper 1 (Th1) cell-mediated immune response in aSyn^−/−^ mice, suggesting a role of α-synuclein for neuroinflammation in EAE and MS.

## Methods

aSyn^−/−^ mice [10] were maintained on a C57BL/6N background for more than 10 generations. All mice were kept under standard animal housing conditions with a 12-h day/night cycle and free access to food and water. For active EAE induction, aSyn^+/+^ (i.e., wildtype C57BL/6N) (Charles River) and aSyn^−/−^ mice received 200 μg myelin oligodendrocyte glycoprotein (MOG)_35–55_ and 200 μg complete Freund’s adjuvant (CFA) subcutaneously. Pertussis toxin (200 ng) was applied intraperitoneally at the time of immunization and 48 h later. Mice were daily weighed and scored for clinical signs using a 10-point scale as described previously [11].

The spinal cord and spleen of aSyn^−/−^ and aSyn^+/+^ mice were removed on day 10 or day 14 after EAE induction. Splenic single-cell suspensions were isolated by enzymatic degradation with DNaseI (10 mg/ml, Roche) and Liberase TL (1.67 Wünsch units/ml, Roche) or by mechanical dissociation [12]. CNS mononuclear cells were prepared by mechanical disruption and subsequent percoll gradient centrifugation as described [12]. Splenocytes and CNS mononuclear cells were analyzed by multicolor flow cytometry using the following fluorochrome-conjugated antibodies: anti-CD3 (17A2), anti-CD4 (RM4-5), anti-CD8a (53-6.7), anti-CD11b (M1/70), anti-CD11c (HL3), anti-CD44 (IM7), anti-CD16/32 (93), anti-CD25 (PC 61.5), anti-CD62L (MEL-14), anti-FoxP3 (FJK-16s), anti-IFN-γ (XMG1.2), and anti-IL-17a (eBio17B7). For intracellular staining, the FoxP3 staining kit (eBioscience) was used. Flow cytometry was performed on a FACS Canto II (BD), and results were analyzed with FlowJo software (Tree Star Inc.).

For lymphocyte proliferation assay, splenic single-cell suspensions were isolated 10 days after EAE induction and stained with proliferation dye eFluor® 450 (eBioscience) according to the manufacturer’s instructions. 1 × 10^6^ cells were seeded in 24-well plates and cultured with media alone or in the presence of 20 μg/ml MOG_35–55_ peptide or 1.25 μg/ml Concanavalin A as control. After 96 h, cells were harvested and processed for flow cytometry analysis.

For in vitro T cell differentiation, splenic T cells were isolated by MACS and re-suspended in MACS buffer at 3 × 10^7^ cells/ml. Sorted naive T cells (CD4^+^CD62L^+^CD44loCD25) were stimulated with 2 μg/ml anti-CD3 and 2 μg/ml anti-CD28. For Th1 cell differentiation, naive T cells were cultured for 4 days with interleukin (IL)-12 (20 ng/ml) and anti-IL-4 (10 mg/ml). For T helper 17 (Th17) cell differentiation, cells were cultured in the presence of IL-6 (40 ng/ml) and rhTGF-β1 (2 ng/ml) for 4 days. For Treg cell differentiation, only rhTGF-β1 (10 ng/ml) was added to culture media.

To evaluate protein levels of α-synuclein, Western blot was performed as previously described [13]. Briefly, total protein was extracted from spinal cord tissue and MACS-purified CD11b^+^, CD11c^+^, and CD4^+^ splenocytes of aSyn^+/+^ and/or aSyn^−/−^ mice either under native conditions or with active EAE prior to symptom onset (10 days after immunization) using radioimmunoprecipitation buffer. α-Synuclein protein was detected by using mouse anti-α-synuclein (Syn1; BD; 610786; 1:300). Mouse anti-β-actin (1:2000; ab8226) was obtained from Abcam. Donkey anti-mouse 488 (Dianova; 1:1000) was used as fluorescence-conjugated secondary antibody. Signal was detected with Fusion FX7 detection system (PeqLab), and densitometric quantifications were performed using the Bio1D software (Vilber Lourmat) by normalizing signals to β-actin.

For real-time polymerase chain reaction (RT-PCR), total RNA was isolated from spleen and spinal cord tissue with the RNeasy Mini Kit (Qiagen) according to the manufacturer’s instructions. Complementary DNA (cDNA) was prepared using the GoScript™ reverse transcription system (Promega). RT-PCR reactions were measured in triplicates either on a LightCycler 480 system (Roche) with the SSO Fast EvaGreen Supermix (Bio-Rad) [13] or on a qTower real-time PCR system (Analytik Jena) [12]. Relative quantification was performed using the ΔΔCT method after normalization to β-actin.

Graphical and statistical analysis was performed with GraphPad Prism® 5 (GraphPad software). Data were analyzed either by ANOVA or Student’s *t* test. Comparison of survival curves (Gehan-Breslow-Wilcoxon) was performed to evaluate the percentage of disease-free mice. Data are presented as mean ± standard error of mean (SEM), and *p < 0.05, **p < 0.01, or ***p < 0.001 was considered to be statistically significant.

## Results

**Fig. 1:**
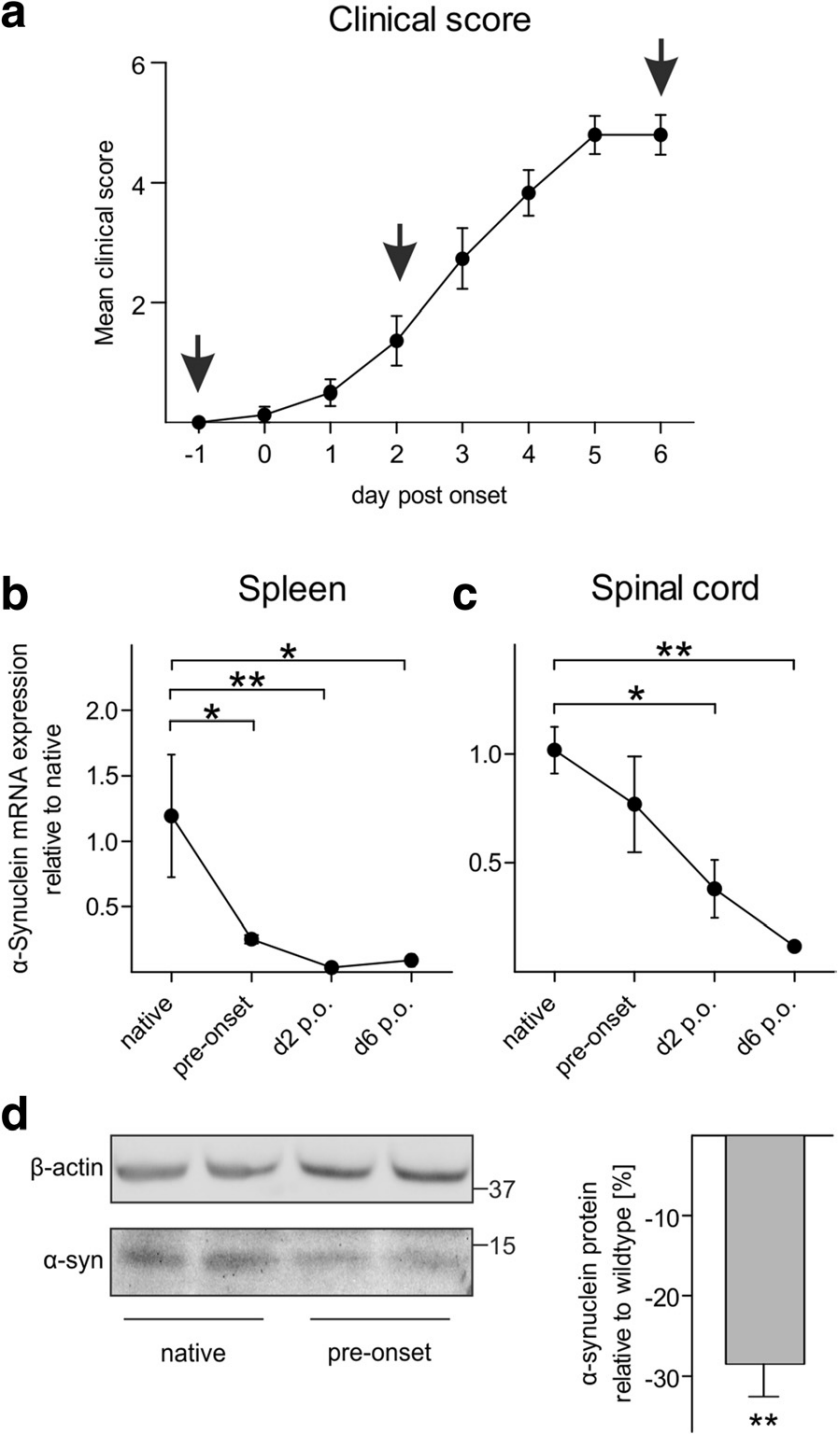
Reduced α-synuclein expression during EAE. **a** Clinical course of MOG_35–55_-induced EAE in C57BL/6N mice. *Arrows* indicate days prior to or post symptom onset on which α-synuclein mRNA expression was analyzed in the spleen (**b**) and spinal cord (**c**). **b**, **c** RT-PCR revealed a gradual downregulation of α-synuclein mRNA expression in the spleen and spinal cord (*n* = 4; *p < 0.05, **p < 0.01). **d** Western blot of spinal cord lysates from EAE mice 10 days after immunization (i.e., prior to symptom onset) demonstrates a significant reduction of α-synuclein (α-syn) protein as compared to the native spinal cord (*n* = 4; **p < 0.01)

We initially examined α-synuclein messenger RNA (mRNA) expression in the spinal cord and spleen in the pre-symptomatic and acute phase of EAE in aSyn^+/+^ (i.e., wildtype C57BL/6N) mice (Fig. 1a). Both in the spleen (Fig. 1b) and spinal cord (Fig. 1c), α-synuclein mRNA expression gradually declined during EAE as compared to native tissues. Reduced α-synuclein mRNA expression was already detected in the pre-symptomatic phase of EAE and reached its lowest expression (reduction by ~90 %) at the peak of disease. Matching the dynamic of α-synuclein gene expression, spinal cord α-synuclein protein levels were significantly reduced by 28 ± 4 % in the pre-symptomatic phase of EAE (Fig. 1d).

**Fig. 2:**
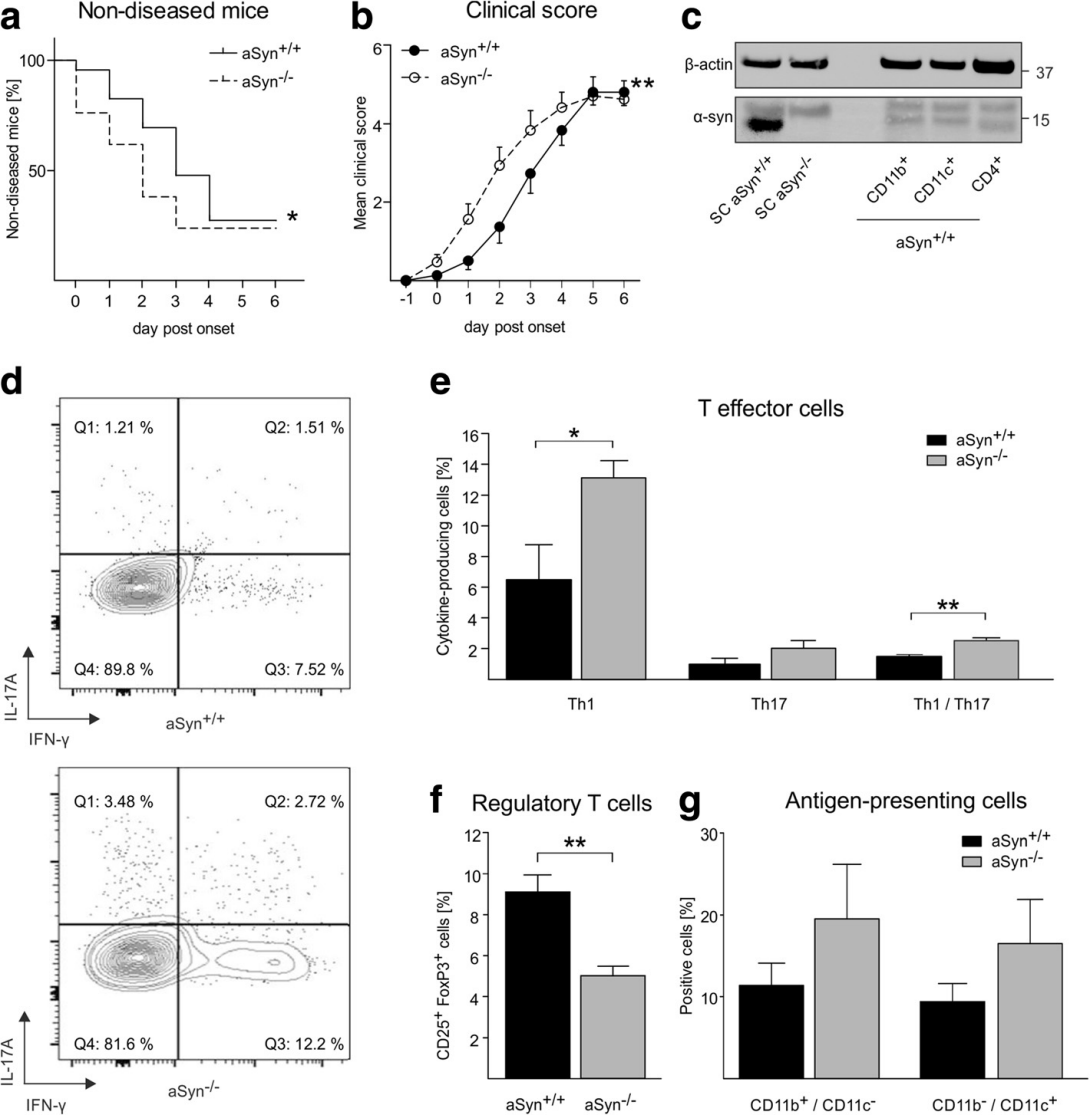
Earlier symptom onset in aSyn^−/−^ mice accompanied by increased spinal cord infiltration of Th1 cells during EAE. **a** Percentage of mice without symptoms during the onset phase of EAE is shown. The aSyn^−/−^ cohort (*dashed line*) exhibited significantly less disease-free mice compared to the aSyn^+/+^ cohort (*solid line*) (*n* = 15/16; *p < 0.05). **b** aSyn^−/−^ mice (*white dots*, *dashed line*) showed a significantly more severe clinical course during the initial phase of EAE than aSyn^+/+^ mice (*black dots*, *solid line*) (*n* = 15/16; **p < 0.01). Symptom severity at disease peak was not different between both groups. **c** Western blot analysis reveals the presence of α-synuclein (α-syn) in antigen-presenting cells (CD11b^+^ and CD11c^+^) and CD4^+^ T cells isolated from the spleen of aSyn^+/+^ mice. Spinal cord (SC) lysates of aSyn^+/+^ and aSyn^−/−^ mice were loaded as positive and negative controls for α-synuclein immunoreactivity. **d** Representative contour plots of ex vivo flow cytometry to determine frequencies of IL-17A^+^ and/or IFN-γ^+^ cells within the spinal cord of aSyn^+/+^ (*upper panel*) and aSyn^−/−^ (*lower panel*) mice 2 days after symptom onset of EAE are depicted. **e** In the spinal cord of aSyn^−/−^ mice (*gray bars*), there were significantly higher frequencies of IFN-γ^+^ Th1 and IFN-γ^+^/IL-17A^+^ Th1/Th17 cells as compared to aSyn^+/+^ mice (*black bars*) (*n* = 4; *p < 0.05, **p < 0.01). **f** Frequency of regulatory T cells was lower in aSyn^−/−^ mice (*n* = 4; **p < 0.01). **g** There was no difference in the frequency of antigen-presenting cells (*n* = 4; *p* > 0.05)

The pre-symptomatic and profound downregulation of α-synuclein expression in EAE suggests that α-synuclein may influence the immune response during the acute phase of EAE. To test this hypothesis, we induced EAE in aSyn^−/−^ and aSyn^+/+^ mice and rated mice for clinical signs during EAE. Unchallenged, aSyn^−/−^ do not display any obvious phenotype and show similar body weights as compared to age-matched aSyn^+/+^ mice. After the induction of EAE, disease onset was significantly earlier (1.2 ± 0.3 days) in aSyn^−/−^ compared to aSyn^+/+^ mice as evidenced by lower number of disease-free mice (Fig. 2a) and a significantly more severe clinical course (Fig. 2b). At the peak of disease, all mice suffered from severe gait ataxia and no difference in clinical scores between aSyn^−/−^ and aSyn^+/+^ mice was observed (Fig. 2b). There were no differences in the overall disease incidence and mortality between both groups.

Symptom onset and severity in EAE are tightly linked to Th1 and/or Th17 cell infiltration into the spinal cord [14]. Moreover, the physiological expression of α-synuclein within antigen-presenting cells (CD11b^+^ or CD11c^+^) and CD4^+^ T cells (Fig. 2c) in wildtype mice suggests that α-synuclein deficiency directly impacts immune responses during EAE in aSyn^−/−^ mice. Thus, to dissect the immunological mechanism responsible for earlier symptom onset in aSyn^−/−^ mice, we next performed ex vivo flow cytometry to determine frequencies of CD4^+^ T cells within the spinal cord (Fig. 2d). Total CD4^+^ cell frequency was twofold but not significantly increased in aSyn^−/−^ spinal cords (aSyn^−/−^ 32.4 ± 8.9, aSyn^+/+^ 14.6 ± 5.1, *p* > 0.05). However, aSyn^−/−^ mice exhibited a significantly increased frequency of interferon gamma (IFN-γ)-producing Th1 cells compared to aSyn^+/+^ mice, whereas IL-17A-producing Th17 cells were not significantly different (Fig. 2e). Notably, T cells that produced both IFN-γ and IL-17A were also significantly increased in aSyn^−/−^ mice (Fig. 2e). Further characterization of infiltrating immune cells revealed a reduced frequency of regulatory T cells in aSyn^−/−^ compared to aSyn^+/+^ mice (Fig. 2f), but neither alterations of CD11b^+^/CD11c^−^ nor of CD11b^−^/CD11c^+^ antigen-presenting cells were detected (Fig. 2g).

**Fig. 3:**
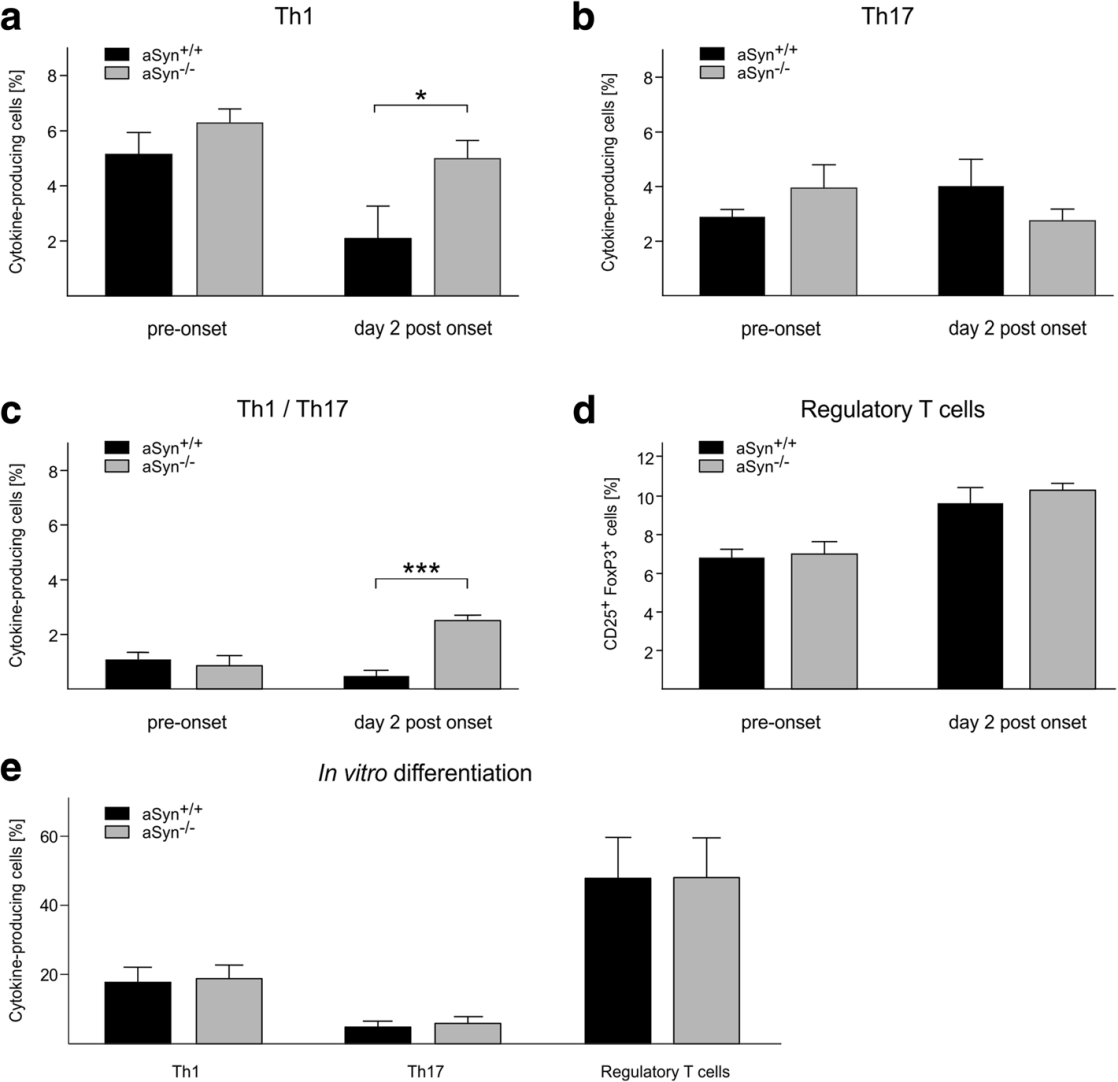
Increased splenic Th1 cell frequency in aSyn^−/−^ mice during the onset phase of EAE. Ex vivo flow cytometry revealed a higher frequency of splenic IFN-γ^+^ Th1 cells 2 days after disease onset in aSyn^−/−^ mice as compared to aSyn^+/+^ mice (*n* = 4; *p < 0.05) (**a**) but no differences in the frequency of IL-17A^+^ Th17 cells (*n* = 4; *p* > 0.05) (**b**). The spleen of aSyn^−/−^ mice also exhibited higher frequencies of IFN-γ^+^/IL-17A^+^ Th1/Th17 cells (*n* = 4; ***p < 0.01) (**c**), whereas regulatory T cells remained unaltered (*n* = 4; *p* > 0.05) (**d**). **e** In vitro differentiation under Th1-, Th17-, or regulatory T cell-polarizing conditions was similar for naive CD4^+^ T cells derived from aSyn^−/−^ and aSyn^+/+^ mice (*n* = 3; *p* > 0.05)

In a next step, we investigated primary immune responses in the periphery by phenotyping splenic CD4^+^ cells prior to (i.e., day 10 post immunization) and 2 days after the onset of symptoms (Fig. 3). Overall, we observed an increased frequency of CD4^+^ T cells in aSyn^−/−^ as compared to aSyn^+/+^ mice. Matching the immunological profile in the spinal cord, a higher frequency of IFN-γ-producing Th1 cells was detected in the spleen of aSyn^−/−^ compared to aSyn^+/+^ mice, particularly 2 days after the occurrence of the first symptoms (Fig. 3a). In line, IL-17A-producing Th17 cells were altered neither prior to nor 2 days after the onset of symptoms (Fig. 3b). Furthermore, T cells producing both IFN-γ and IL-17A were also significantly increased in the spleen of aSyn^−/−^ mice but only after symptom onset (Fig. 3c). There were no differences in the frequency of regulatory T cells (Fig. 3d). In parallel, α-synuclein deficiency resulted in an increase of activated splenic effector T cells prior to symptom onset with significantly increased CD4^+^/CD44^high^ (aSyn^−/−^ 52.13 ± 0.82, aSyn^+/+^ 48.27 ± 1.27; *p* < 0.05) and CD4^+^/CD25^high^ cell frequency (aSyn^−/−^ 55.03 ± 1.03, aSyn^+/+^ 51.60 ± 0.99; *p* < 0.05). An in vitro T cell differentiation assay with splenic T cells revealed no difference regarding the differentiation into T helper cell subtypes (i.e., Th1, Th17, regulatory T cells) between aSyn^−/−^ mice and controls (Fig. 3e).

**Fig. 4:**
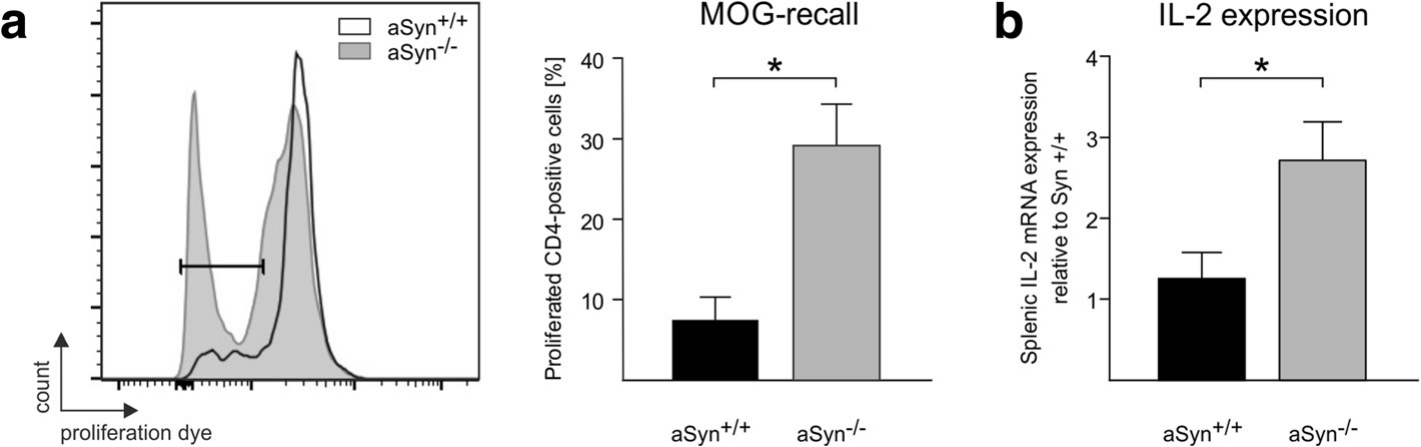
Increased proliferation of α-synuclein-deficient splenocytes associated with increased splenic IL-2 mRNA expression in aSyn^−/−^ mice prior to onset of EAE. **a** Proliferation of CD4^+^ cells was measured by flow cytometry in splenocyte cultures after ex vivo recall with MOG_35–55_ (splenocytes harvested on day 10 post immunization in aSyn^−/−^ and aSyn^+/+^ mice), revealing significantly increased proliferation of aSyn^−/−^-derived splenocytes (*n* = 4; *p < 0.05). **b** RT-PCR was performed on cDNA derived from total RNA that was isolated from the spleen of aSyn^−/−^ and aSyn^+/+^ mice 10 days after induction of EAE. IL-2 mRNA expression was significantly increased in the spleen of aSyn^−/−^ mice (*n* = 4; *p < 0.05)

In line with the increased frequency of splenic CD4^+^ T cells, CD4^+^ splenocytes isolated from aSyn^−/−^ mice showed enhanced proliferation upon ex vivo re-stimulation with MOG_35–55_ peptide as compared to aSyn^+/+^ splenocytes (Fig. 4a). Consisting with this hyperproliferative phenotype of aSyn^−/−^ splenocytes, the levels of IL-2 mRNA were significantly increased in the spleen of aSyn^−/−^ mice in EAE prior to symptom onset (Fig. 4b), whereas IL-4 expression was unaltered (aSyn^−/−^ 1.20 ± 0.37, aSyn^+/+^ 1.44 ± 0,41; *p* > 0.05).

## Discussion

Our study reveals that α-synuclein expression gradually declines during EAE of C57BL/6N mice, beginning already in the pre-symptomatic phase. Furthermore, EAE in aSyn^−/−^ mice is characterized by earlier symptom onset and enhanced T cell—in particular Th1 cell—infiltration into the spinal cord, consisting of higher splenic T cell frequencies and increased T cell proliferation.

Our data on the role of α-synuclein during the acute phase of EAE extends previous descriptive work on α-synuclein in MS and EAE. In demyelinated MS lesions with active inflammation, but not in chronic inactive lesions, increased α-synuclein immunoreactivity has been observed in microglia/macrophages as well as in neurons and oligodendrocytes [9]. Interestingly, elevated α-synuclein expression in neurons and glia during MOG-induced EAE in rats has been correlated with degree of inflammation and demyelination [15]. In a small study investigating cerebrospinal fluid, α-synuclein concentrations were significantly higher in patients with inflammatory demyelinating disease than in patients suffering from PD or controls [16].

The phenotypical characterization of T cells within the spinal cord at the onset of EAE showed increased Th1 cell infiltration in aSyn^−/−^ mice. Matching this finding, the spleen of aSyn^−/−^ mice also exhibited higher frequencies of Th1 cells. Furthermore, increased spinal cord Th1 cell infiltration was accompanied by lower frequencies of anti-inflammatory neuroprotective regulatory T cells, whereas Th17 and antigen-presenting cells remained unaffected. These data suggest that α-synuclein deficiency promotes Th1 cell activation and negatively impacts regulatory T cells, thereby exacerbating Th1-mediated immune response in EAE. In agreement with this interpretation, a functional role of α-synuclein for T cell activation has previously been suggested as splenic CD4^+^ cells isolated from α-synuclein-deficient mice showed increased activity [17]. Moreover, increased CNS regulatory T cell frequency has been observed upon immunization with nitrated α-synuclein protein in the 1-methyl-4-phenyl-1,2,3,6-tetrahydropyridine (MPTP) model of PD [18].

Results from splenocyte recall assays on day 10 after EAE induction imply that enhanced T cell proliferation underlies expansion of Th1 cells in aSyn^−/−^ mice. In good agreement with this finding, we observed increased mRNA expression of IL-2 as central activator of T cell proliferation [19] in the spleen of aSyn^−/−^ mice on day 10 after EAE induction, whereas mRNA expression of IL-4 as key cytokine during Th2 cell-mediated immune response was unaltered. These data suggest that α-synuclein deficiency leads to increased IL-2 production, thereby expanding the population of Th1 cells. In line with this interpretation, immunization with nitrated α-synuclein protein in the MPTP model of PD elicits decreased Th1 cell-mediated inflammation and reduction of IL-2 levels [18].

Overall, our data show that endogenous α-synuclein plays a functional role for immunological processes during early EAE as a new regulator of Th1 responses in neuroinflammation. Hence, our study identifies α-synuclein as an interesting new target for modulating the immune response in MS.

## Abbreviations

aSyn^+/+^: Wildtype C57BL/6N
aSyn^−/−^: α-Synuclein knockout
CFA: Complete Freund’s adjuvant
CNS: Central nervous system
EAE: Experimental autoimmune encephalomyelitis
IFN-γ: Interferon gamma
IL: Interleukin
MOG: Myelin oligodendrocyte glycoprotein
MPTP: 1-Methyl-4-phenyl-1,2,3,6-tetrahydropyridine
MS: Multiple sclerosis
PD: Parkinson’s disease
RT-PCR: Real-time polymerase chain reaction
Th1: T helper 1
Th17: T helper 17
